# Subtypes of Alcoholics Based on Psychometric Measures

**Published:** 1996

**Authors:** John P. Allen

**Affiliations:** John P. Allen, Ph.D., is chief of the Treatment Research Branch, National Institute on Alcohol Abuse and Alcoholism, Bethesda, Maryland

**Keywords:** AOD dependence, disorder classification, psychological assessment, personality test, motivation, treatment, comorbidity, patient monitoring

## Abstract

One approach to subtyping alcoholics is the use of psychometric tests that quantify a person’s personality characteristics, psychological characteristics, and intelligence. For example, researchers have used the Personality Research Form, which measures basic personality traits, to establish alcoholism typologies. Other psychometric measures that have been employed in the classification of alcoholics, such as the Minnesota Multiphasic Personality Inventory and the Millon Clinical Multiaxial Inventory, measure the presence of co-occurring psychiatric disorders in the patients. Still other subtypes are based on tests assessing the patient’s motivation for treatment. Although clinicians hope to use psychometric typologies to improve treatment planning and monitoring for their patients, several questions remain to be answered by additional research before the instruments and the typologies based on them achieve broad applicability.

Alcoholics differ significantly with respect to their personality types, their patterns of alcohol consumption, and the kind and severity of their drinking problems. Similarly, a wide variety of interventions exist for treating alcoholism. A major goal of contemporary alcoholism research is to develop decision rules allowing treatment providers to assign patients to specific interventions that are most effective for them. Because it is not feasible to design a personalized treatment program for each alcoholic, a prerequisite for such decision rules is the identification of subtypes of alcoholics that share common characteristics.

Researchers have pursued many different approaches to developing alcoholism typologies (for more information, see the article by Babor, pp. 6–14). To have meaningful implications for alcoholism treatment, typologies should meet at least the following five criteria:

- The measure used to assign patients to subtypes is easy to administer and evaluate.
- The vast majority of patients can be classified into a relatively small number of distinct subtypes.
- The subtyping measure can be administered early during the course of treatment so that the most appropriate intervention(s) can be selected.
- The typology allows treatment providers to select the appropriate treatment approach available within their programs or to suggest appropriate referral options for needed services not available within the programs.
- The results of the subtyping measure provide feedback to the patients and enhance their motivation for treatment.

One approach to assessing and subtyping alcoholics is the use of psychometric measures, that is, tests that quantify a person’s personality characteristics, psychological characteristics, or intelligence. These instruments, which usually are questionnaires, have several advantages over more subjective assessment strategies, such as clinical interviews. For most psychometric tests, researchers already have determined their validity (i.e., that the tests actually measure the characteristics they are supposed to measure) and reliability (i.e., that the results are reproducible). Furthermore, existing standards show how the results of a given patient differ from his or her peers. Psychometric measures also can evaluate domains of interest (e.g., the presence of certain personality characteristics) with greater comprehensiveness and less bias than other assessment tools. Finally, these tests tend to be more economical, because their completion by the patient does not require the presence of a clinician, and many appropriate instruments either are not copyrighted or may be used after paying only a small royalty.

This article reviews systems for subtyping alcoholics in treatment based on cluster analyses^1^ of psychometric tests that assess the alcoholic’s personality traits, psychopathology, or treatment motivation. Most of these typologies are based on a relatively small number of tests, including the Personality Research Form (PRF), the Minnesota Multiphasic Personality Inventory (MMPI), and the Millon Clinical Multiaxial Inventory (MCMI), which are discussed below. However, other assessment measures (e.g., the Alcohol Use Inventory [Donat 1994], the Michigan Alcoholism Screening Test [Snowden et al. 1986], and cognitive-neuropsychological measures [Donovan et al. 1986]) have been used occasionally. This article also highlights some of the questions that must be addressed before the clinical utility of these psychometric measures and typologies can be determined unequivocally.

## Personality-Based Typologies

Approaches to classifying alcoholics according to their personality characteristics (e.g., impulsivity and emotionality) are based on the assumption that such characteristics could influence both the risk for and the treatment of alcohol and other drug (AOD) abuse. One of the most technically sophisticated and highly researched measures of basic personality traits is the PRF (Jackson 1984). This test assesses 20 common human needs, such as achievement and social recognition, rather than characteristics that reflect major emotional problems, such as depression or schizophrenia. The test has been used extensively to determine risk factors for AOD abuse and personality characteristics associated with the treatment process (Allen et al. 1994).

Several researchers have used the PRF to develop typologies of alcoholics in treatment (Nerviano 1976, 1981; Zivich 1981). Although the sample characteristics and specific PRF versions used differed slightly across these studies, their results seem largely consistent. In all three studies, the investigators identified several subtypes that differed not only in their characteristics according to the PRF but also with respect to characteristics assessed by other psychometric instruments (e.g., the MMPI). The relevance of these studies, however, is somewhat limited because they included only male alcoholics. Furthermore, the researchers could assign only approximately 50 percent of the subjects unambiguously to any of the subtypes, a percentage lower than that of most other subtyping systems.

The PRF also served as the basis for alcoholism subtypes in an investigation by Allen and colleagues (1994) that included both male and female AOD-dependent inpatients. The researchers distinguished five subtypes, which encompassed almost all the subjects. The gender composition, however, varied significantly among the subtypes. As in the earlier studies, the subtypes differed not only in the characteristics assessed by the PRF but also in two independent MMPI measures related to the expression of impulses and emotionality. Moreover, subtype membership correlated with the likelihood that certain internal and external stimuli prompted AOD use. The researchers proposed that differences among the subtypes regarding measures of psychopathology and stimuli for AOD abuse could have implications for the most effective type of intervention for each subtype. The actual value of this theoretical “patient-treatment matching” approach, however, has not yet been determined.

## Psychopathology-Based Typologies

High rates of co-occurring psychiatric disorders (e.g., antisocial personality disorder, depression, and schizophrenia) among patients in alcoholism treatment suggest that meaningful subtypes could be defined based on the presence or absence of these disorders. Two psychometric instruments, the MMPI and the MCMI, have proven especially valuable in this regard.

**Figure:**
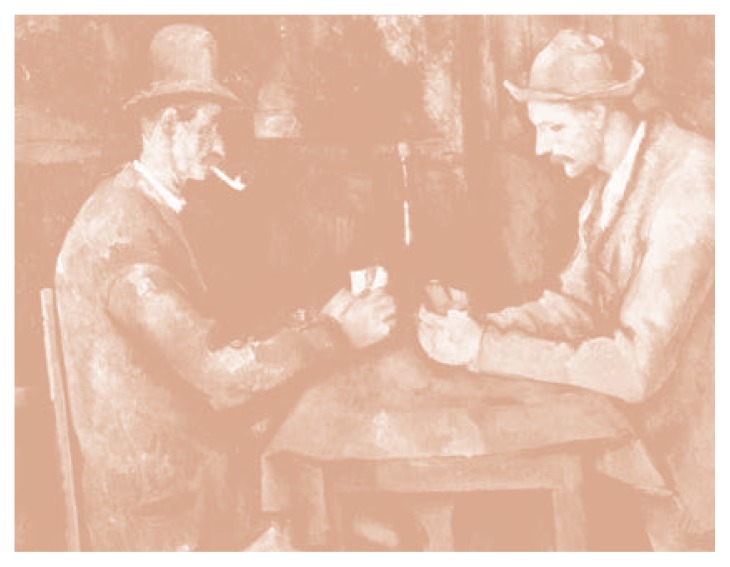
Type I/type A alcoholism illustrated in “Les joueurs de cartes,” 1890–1895, by Paul Cézanne. Reproduced with permission from the Musée du Jeu de Paume. © des Musées Nationaux, Agence Photo RMN.

### Typologies Based on the MMPI

The MMPI is by far the most frequently used psychometric measure for developing typologies of alcoholics in treatment. This test, which consists of 550 questions, is used frequently to determine personality characteristics and to diagnose various psychopathological disorders. The MMPI questions can be grouped into different scales, or sets of questions that focus on specific aspects of personality or psychopathology (e.g., impulsivity or depression). When interpreting the test results, clinicians focus primarily on the pattern of scales on which a patient scores highest, rather than on the specific scores on each scale. Accordingly, a test result is expressed as a profile code (e.g., 2–8–7–4) according to the scales on which the patient exhibited the highest scores. (For more information on the MMPI, see the article by Ingle, pp. 63–66.)

Several studies have resulted in typologies based on MMPI test performance (e.g., Graham and Strenger 1988; Morey et al. 1987). Although these studies generally differed in the number and characteristics of subtypes, they consistently identified at least one alcoholic subtype with prominently elevated scores on scale 4, a scale measuring psychopathy. These elevated scale 4 scores declined only slightly and remained above normal during alcoholism treatment (Graham and Strenger 1988). The significance of this scale for evaluating alcoholics is underscored by findings that elevated scale 4 scores also could predict future alcohol problems in young male college students long before they were likely to suffer from alcohol problems or enter alcoholism treatment (Kammeier et al. 1973).

Several researchers attempted to consolidate the various MMPI-based typologies reported in the literature by comparing subtypes described across a wide range of studies. As a result of such analyses, Graham and Strenger (1988) suggested that six reproducible subtypes of alcoholics existed, which differed on several personality, psychopathological, drinking, and treatment-related variables. In contrast, Morey and colleagues (1987) postulated only three subtypes (i.e., early stage problem drinkers, affiliative alcoholics, and schizoid alcoholics^2^) in an MMPI-based typology. These three subtypes originally had been identified in the researchers’ “hybrid model” (described later) and were based primarily on the Alcohol Use Inventory^3^ (Morey et al. 1984). The researchers found that of the 79 MMPI subtype profiles from 11 studies analyzed, 91 percent correlated significantly with 1 or more of the 3 subtypes. All three subtypes were characterized by a 2–8–7–4 MMPI profile code, indicating elevated scores on scales that reflect high degrees of depression, alienation from others, unusual thought patterns, anxiety, and impulsivity. Differences between the subtypes existed mainly in the degree of elevation on these scales.

To assess the usefulness of MMPI-based typologies in determining adequate treatment for alcoholics and in evaluating treatment results, researchers also examined the stability of MMPI scores during the course of alcoholism treatment. These studies found that over 30 days of treatment, the overall elevation of clinical scales tended to decline and the profiles became less distinctive (e.g., Dush and Keen 1995; Sheppard et al. 1988). Moreover, some subjects fell into a different subtype after repeated testing. These findings suggest that not only is MMPI-based subtyping helpful in initial treatment planning but that repeating the test during treatment could assist in planning later treatment stages. Alternatively, the changes in MMPI scores and subtype affiliation could indicate that MMPI scores determined during withdrawal bear little relation to the person’s MMPI scores before the onset of alcoholism, which would be more relevant for treatment. Both of these interpretations suggest, however, that MMPI testing probably should be delayed until the patient’s condition has stabilized after detoxification.

### Typologies Based on the MCMI

The MCMI (Millon 1983) is a 175-item survey that assesses the psychological characteristics of psychiatric patients. The test has 20 scales that evaluate the patients with respect to 8 basic personality styles (e.g., how the subjects relate with other people), 3 severe personality disorders, and 9 classes of acute symptoms of emotional difficulties.

Four studies defined subtypes of AOD-abusing patients based on MCMI analyses (Bartsch and Hoffman 1985; Donat 1988; Mayer and Scott 1988; Donat et al. 1991). The results of these studies were as follows (the studies did not actually label the subtypes but described them based on MCMI scales most distinctive for each subtype):

- Bartsch and Hoffman (1985) identified five subtypes among a sample of male Veterans Affairs (VA) clients in inpatient alcoholism treatment.
- Donat (1988) distinguished five subtypes in a sample including both women and men. These subtypes appear to correspond to those proposed by Bartsch and Hoffman (1985) based on a comparison of the patterns of MCMI scales with high scores between the two studies. Gender had only a minor effect on subtype membership: Although women were overrepresented in one category and underrepresented in another, these two subtypes had similar profiles of MCMI characteristics.
- Mayer and Scott (1988) assessed male, alcoholic VA inpatients using only the MCMI scales pertaining to personality styles and severe personality disorders. The patients fell into four subtypes that differed primarily with respect to psychological difficulties (e.g., hallucinations, suicide attempts, and psychiatric hospitalizations). Pairs of subtypes also differed on several drinking-related variables, such as age of onset of alcohol abuse, presence of withdrawal seizures, and likelihood of completing treatment. Three of the subtypes corresponded to those identified by Bartsch and Hoffman (1985), and the fourth subtype was moderately related to one of the remaining two subtypes.
- Donat and colleagues (1991) also divided their male and female subjects into five subtypes. These categories differed on several scales of the Alcohol Use Inventory that pertained to perceived benefits of drinking, problems resulting from drinking, and—to a lesser extent—style of drinking (e.g., drinking alone or with others). The researchers also compared the mean scores on the MCMI scales for their five subtypes and those proposed by Bartsch and Hoffman (1985) and Mayer and Scott (1988). These analyses indicated a high degree of correspondence between the subtypes derived from all three studies.

## Subtyping Based on Motivation for Treatment

The effectiveness of interventions during alcoholism treatment may depend on the patients’ motivation for treatment. For example, people who do not realize that they have an alcohol problem might do better with interventions designed to help them acknowledge the need for change. In contrast, alcoholics who already are aware of their problem may respond better to treatment focusing on how to make personal changes. Consequently, subtyping patients based on their readiness to change may have considerable clinical value.

The University of Rhode Island Change Assessment Scale (URICA) (McConnaughy et al. 1983) is a popular approach to evaluating patient motivation. Although this scale has been used predominantly in research on smoking cessation, it also recently has been employed in alcoholism treatment studies. The URICA is a brief self-report measure that scores patients on four scales representing stages of motivation for change (Prochaska and DiClemente 1986): (1) precontemplation (i.e., unawareness of the need to change one’s drinking behavior), (2) contemplation (i.e., acknowledging the problem and seriously considering necessary changes), (3) action (i.e., engaging in concrete efforts to change and seeking assistance), and (4) maintenance (i.e., attempting to consolidate and sustain positive gains achieved). Although most patients fall primarily into one category in this change process, they may display behaviors and express attitudes associated with an additional—usually adjacent—stage.

Two studies examined a large number of patients entering treatment for AOD abuse with respect to their URICA stage patterns (DiClemente and Hughes 1990; Carney and Kivlahan 1995). Although their samples differed in age, gender, diagnosis, and employment patterns, both studies identified four similar subtypes that they labeled precontemplation, contemplation, participation, and ambivalent. Subjects of the precontemplation subtype had high scores on the URICA precontemplation scale and low scores on the contemplation scale. The contemplation subtype had high scores on the contemplation scale and low scores on both the precontemplation and action scales. Finally, the ambivalent subtype, like the precontemplation subtype, scored high on the precontemplation scale but also had moderate scores on the scales representing the other three stages of change. In addition, DiClemente and Hughes (1990) identified a subtype they labeled as uninvolved or discouraged, which included patients with low scores on all URICA scales. Patients in the various subtypes differed with respect to the perceived benefits, styles, consequences, and concerns related to drinking; the perceived ability to change their own behavior; and the extent to which they were tempted to drink.

An alternative measure of treatment motivation is the Stages of Change Readiness and Treatment Eagerness Scale (Miller 1992). In one study, cluster analysis of the outcome of male, AOD-abusing VA inpatients using this scale indicated that the patients fell into three subtypes labeled ambivalent, uninvolved, and active (Isenhart 1994). Seventy percent of the subjects in this study belonged to the active subtype. The greatest differences predictably existed between the uninvolved and active subtypes. Compared with members of the uninvolved subtype, members of the active subtype exhibited higher levels of alcohol use, involvement with and dependence on alcohol, loss of control over drinking, and awareness of adverse consequences of drinking. The three subtypes, however, did not differ in their MMPI profiles or with respect to sociodemographic factors.

## The Hybrid Model for Classifying Alcoholism

Most alcoholism typologies based on psychometric measures assess only one domain (e.g., personality, psychopathology, or treatment motivation). Some researchers, however, have attempted to classify alcoholics simultaneously by the type and severity of their problems and by multiple underlying patient characteristics. The best elaborated of these schemes was developed by Morey and colleagues (1984), who distinguished three subtypes of alcoholics based primarily on subscale scores of the Alcohol Use Inventory. These subtypes—which included early stage problem drinkers, affiliative alcoholics, and schizoid alcoholics—differed on several dimensions, including personality traits (as assessed by the PRF), intellectual functioning, demographics, psychopathology, and alcohol use, as follows:

- Early stage problem drinkers reported later onset of drinking, drank less per day, and suffered fewer adverse consequences due to drinking than members of the other subtypes. In addition, the early stage problem drinkers differed from patients in the other subtypes by exhibiting higher needs for achievement and abstract thinking as well as reduced levels of aggressiveness and impulsivity. Overall, these patients’ characteristics were relatively close to the norms established in the PRF.
- Affiliative alcoholics tended to drink more continuously than early stage problem drinkers. In addition, compared with the other subtypes, the affiliative alcoholics also were more likely to drink with others, were more heavily influenced by peers, and reported more interpersonal difficulties.
- Schizoid alcoholics revealed the most severe drinking problems and drinking consequences and frequently suffered from anxiety and feelings of guilt. In contrast to the affiliative alcoholics, they typically drank alone and tended to engage in binge drinking rather than continuous drinking. Compared with the other two subtypes, schizoid alcoholics had higher PRF scores indicative of aggression and impulsivity and lower scores on traits of affiliation and understanding.

Proponents of the hybrid model have attempted to correlate these three subtypes with other existing alcohol typologies. These analyses indicated that the affiliative alcoholic subtype had some similarities with Jellinek’s delta alcoholism (Morey and Skinner 1986), Cloninger’s type I alcoholics^4^ (Morey and Jones 1992), and Babor’s type A variant of alcoholism (Babor et al. 1992). Conversely, the schizoid alcoholic subtype resembled Jellinek’s gamma alcoholism (Morey and Skinner 1986), Cloninger’s type II alcoholism (Morey and Jones 1992), and Babor’s type B variant (Babor et al. 1992). (For more information on the typologies of Jellinek, Cloninger, and Babor, see the article by Babor, pp. 6–14.)

## Usefulness of Psychometric Typologies for Treatment Planning

As mentioned previously, to provide meaningful implications for treatment planning, typologies should meet at least five requirements. All the psychometric instruments and typologies discussed here satisfy at least two of these criteria: (1) they are relatively easy to administer and (2) they classify the majority of patients into a limited number of subtypes. However, research has not yet adequately addressed whether these psychometric tests can be administered and reliably evaluated early in treatment. Clinicians generally delay testing until patients have stabilized, often 7 to 10 days after entering treatment. To date, no clinical studies have determined the degree of cognitive and emotional stabilization required for the results of various classes of personality tests to be considered valid.

Similarly, no published studies have rigorously examined how the various subtypes described by psychometric typologies respond to different treatment alternatives and whether such typologies lead to more effective assignment of patients to treatment. Many reports on subtypes offer treatment recommendations, and general research on enhancing treatment outcome by matching patients to interventions based on particular needs has yielded positive results (Mattson et al. 1994). The extent to which psychometrically based typologies actually improve clinicians’ abilities to plan alcoholism treatment, however, has not been determined.

With respect to satisfying the requirement that the results of subtyping enhance the patient’s motivation for treatment, alcohol-specific typologies (e.g., the hybrid model) probably are more effective than systems based on general personality characteristics or co-occurring psychopathology. The latter two domains are likely more difficult to change through treatment or patient choice than are drinking patterns or the motivation for rehabilitation.

## Needs for Further Research

Although extensive research has examined the use of psychometric instruments to classify alcoholic patients into specific subtypes, several salient questions remain to be answered.

First, and most important, researchers must evaluate the applied value of alcoholism typologies. This includes determining the extent to which assigning patients to treatment interventions based on subtype improves outcome, compared with random assignment or assignment according to other systems, such as patient gender, severity of the patient’s problems, or practical convenience of the intervention.

Second, little research has evaluated similarities of psychometric typologies using the same test across studies or employing different tests within studies. Although the visual appearance of psychometric profiles or verbal descriptions of subtypes may appear similar in different studies, the actual congruence between subtypes must be determined through statistical analyses (Bohn and Meyer 1994). Unfortunately, this has rarely been done.

Third, researchers should evaluate the potential of additional psychometric measures for classifying alcoholics in treatment. The prospects for deriving useful alcoholism typologies from such tests depend on both the relationship of patient variables to available treatment choices and on the tests’ accuracy in assessing these variables. Tests failing to measure treatment-relevant variables accurately are unlikely to yield workable typologies. Several measures exist, however, that may have strong implications for alcoholism treatment planning and thus could prove useful for classifying alcoholics. These include the Alcohol Expectancy Questionnaire (Brown et al. 1987) and the Inventory of Drinking Situations (Annis 1982) as well as robust measures of broad personality characteristics, such as the Neuroticism Extraversion and Openness Personality Inventory (NEO) (Briggs 1992). To date, these instruments have not yet been used to develop alcoholism typologies.

Fourth, the possible influence of gender on subtyping should be explored in more detail. Although some of the studies mentioned here have observed differences in the gender composition of certain subtypes, the potential effects of gender on the fundamental structure of a psychometrically based typology have not been studied. Such studies would require researchers to develop separate typologies for male and female samples and compare the resulting classification systems. Similarly, little is known about psychometric typologies of adolescents entering alcoholism treatment (Massey et al. 1992).

Fifth, investigators have not yet addressed the correlation between the choice of typology instruments used in a given treatment program and the treatment options available in that program. It seems reasonable to assume that for optimal treatment results, the psychometric instrument chosen in a facility would be related to the range of available treatment possibilities. For example, instruments and typologies based on measures of psychopathology would be expected to be most helpful in programs equipped to treat patients with co-occurring psychiatric disorders. Similarly, typologies reflecting the patient’s readiness for change might prove most useful in behaviorally focused treatment programs with components available to enhance or sustain treatment motivation.

Finally, it would be of interest to compare typologies of alcoholic patients with those of matched patients with other behavioral or psychiatric problems or of healthy people using the same psychometric measure. Such comparisons could increase our knowledge of the effects of alcohol problems on other basic dimensions of functioning and enhance our understanding of alcoholic patients’ treatment needs by comparing them with other clinical populations.

## Outlook on Treatment Planning

One of the potential benefits of developing typologies is to allow treatment providers to quickly and easily assign alcoholics to patient groups with similar treatment needs. So far, researchers do not know whether—and to what extent—existing psychometric typologies have achieved this goal. In addition, treatment providers can base their treatment decisions on alternative measures, such as non-test-based typologies, many of which are described elsewhere in this journal issue, thereby further confounding the relevance of psychometric typologies. Another treatment-planning strategy could involve a “menu-driven” approach in which patients are assigned to a variety of specific treatment modules related to their individual needs beyond those indicated by their alcoholism subtype affiliation. Furthermore, researchers in the future may develop a particularly potent medication or behavioral intervention that can benefit all alcoholic patients regardless of their subtype. Thus, although typologies based on psychometric measures offer intriguing suggestions for more effectively determining the needs of alcoholics in treatment, only well-controlled research can demonstrate the value of these and other strategies in improving the treatment outcome of alcoholics.

## Footnotes

1 Cluster analysis is a statistical technique that groups individuals into a small number of subtypes based on similarities of members’ scores on several variables. The technique also attempts to establish subtypes that differ considerably from one another.

2 These subtypes are described in more detail on p. 27.

3 The Alcohol Use Inventory is a self-administered test that assesses drinking behavior and frequently is used in treatment planning.

4 In contrast to Cloninger’s typology, however, both the affiliative and schizoid alcoholics exhibit an early onset of drinking problems and do not differ in their gender composition or in their family history of alcoholism.
